# Camphor and Hydrate Chloral

**Published:** 1881-11-20

**Authors:** 


### CAMPHOR AND HYDRATE CHLORAL.

M. Simons having observed a case of poisoning by a mixture of equal parts camphor and hydrate chloral, conceived the idea of employing the same preparation in therapeutic doses. Twenty drops of this mixture in a draught cut short an attack of acute mania. M. Simons believes that it could be employed with good results in hydrophobia, tetanus, and delirium tremens.—*Med. Press and Circ.*, Sept. 7. —*N. Y. Medical Abstract.*

